# Knowledge mapping and research hotspots of immunotherapy in renal cell carcinoma: A text-mining study from 2002 to 2021

**DOI:** 10.3389/fimmu.2022.969217

**Published:** 2022-07-28

**Authors:** Kun Liu, Seling Zhao, Jian Li, Yikun Zheng, Haiyang Wu, Jianqiu Kong, Zefeng Shen

**Affiliations:** ^1^ Department of Urology, Xiamen Humanity Hospital, Fujian Medical University, Xiamen, China; ^2^ Graduate School of Tianjin Medical University, Tianjin, China; ^3^ Department of Urology, Sun Yat-sen Memorial Hospital, Sun Yat-sen University, Guangzhou, China; ^4^ Guangdong Provincial Key Laboratory of Malignant Tumor Epigenetics and Gene Regulation, Guangzhou, China

**Keywords:** immunotherapy, renal cell carcinoma, immune checkpoint inhibitors, bibliometric, visualization

## Abstract

**Background:**

Renal cell carcinoma (RCC) is one of the most lethal urological malignancies, and because early-stage RCC is asymptomatic, many patients present metastatic diseases at first diagnosis. With the development of immunotherapy, the treatment of RCC has entered a new stage and has made a series of progress. This study mainly outlines the knowledge map and detects the potential research hotspots by using bibliometric analysis.

**Methods:**

Publications concerning RCC immunotherapy from 2002 to 2021 in the Web of Science Core Collection were collected. Visualization and statistical analysis were mainly performed by freeware tools VOSviewer, CiteSpace, R software, and Microsoft Office Excel 2019.

**Results:**

A total of 3,432 papers were collected in this study, and the annual number of papers and citations showed a steady growth trend. The United States is the leading country with the most high-quality publications and is also the country with the most international cooperation. The University of Texas MD Anderson Cancer Center is the most productive organization. The *Journal of Clinical Oncology* is the highest co-cited journal, and Brian I. Rini is both the most prolific author and the author with the largest centrality. The current research hotspots may be focused on “immune checkpoint inhibitors (ICIs),” “PD-1,” and “mammalian target of rapamycin.”

**Conclusion:**

Immunotherapy has a bright future in the field of RCC treatment, among which ICIs are one of the most important research hotspots. The main future research directions of ICI-based immunotherapy may focus on combination therapy, ICI monotherapy, and the development of new predictive biomarkers.

## Introduction

Renal cell carcinoma (RCC) is a common malignant tumor in the genitourinary system and one of the top 10 most common cancers in the world (1–3). More than 60% of patients with RCC suffer from localized tumors (4, 5). However, more than 15% of them may develop locally advanced progression. In addition, although early-stage RCC is curable, it is usually hard to detect, as patients are usually asymptomatic or mildly symptomatic. Many studies demonstrated that 16% of patients presented metastatic diseases at first diagnosis (6). Currently, surgical removal remains the mainstay of the treatment of RCC at an early stage, and after effective surgical treatment, the 5-year overall survival (OS) can exceed 90% (7). Unfortunately, the 5-year OS will decrease to 12% once metastatic disease occurs (8). Moreover, RCC is poorly responsive to conventional chemotherapy and radiotherapy, which leads to poor prognosis in patients with metastatic disease (9–11).

The emergence and development of immunotherapy provide more therapeutic methods for patients with RCC. Immunotherapy, as a potentially beneficial addition to conventional treatments for cancers, can modulate and enhance the host’s immune system to eliminate tumor cells and prevent tumor recurrence so as to prolong the survival times of patients (12). The emergence of treatment targeting immune checkpoints signaled the arrival of the era of immunotherapy (13). After a long period of development, cancer immunotherapy has revolutionized oncology and provides new treatment options for many refractory tumors. At present, the most common cancer immunotherapy methods are adoptive T-cell therapy and chimeric antigen receptor T-cell immunotherapy (CAR-T) (14). Findings from previous studies showed that CAR-T has been widely applied in the treatment of multiple tumors, including B-cell acute lymphoblastic leukemia, and has achieved therapeutic effects. However, no significant therapeutic effects were found on solid tumors (15–17).

Over the past few decades, benefiting from the development of vascular endothelial growth factor (VEGF) tyrosine kinase inhibitors and immune checkpoint inhibitors (ICIs), immunotherapy has been widely used in the treatment of RCC (18–20). As of 2018, ipilimumab plus nivolumab was the only ICI drug treatment approved by the Food and Drug Administration (FDA) for the treatment of RCC (21). Currently, a variety of ICIs have been approved by the FDA, and the combination of ICIs and tyrosine kinase inhibitors is the first-line treatment of clear cell RCC (ccRCC) (22). Moreover, ICI monotherapy is currently undergoing clinical trials and is expected to become a promising alternative therapeutic method for patients with combination therapy intolerance (23–25). The research focus in the field of RCC immunotherapy is constantly changing with the effect of drugs, indicating that the research topics related to cancer immunotherapy are being updated rapidly, and it is necessary to monitor the research progress. Therefore, determining the current research hotspots and future research trends in this field may contribute to understanding the latest research directions.

Bibliometrics uses the citation data from database to evaluate the published research and to systematically study and visualize the knowledge structure and development trend of a certain scientific field by qualitatively and quantitatively analyzing the cooperation, co-occurrence, or co-citation of publications (26–29). It is a powerful tool to investigate the progress of research on different topics and to assess future research trends. Currently, bibliometric analysis has been applied in many fields (30, 31), but there is no specific bibliometric study in the field of RCC immunotherapy. The purpose of this study is to create a comprehensive summary of existing publications on RCC immunotherapy research in the past 20 years through bibliometrics, aiming to perform knowledge mapping to explore the hotspots or frontiers in this field.

## Methods

### Database and study collection

**Figure 1:**
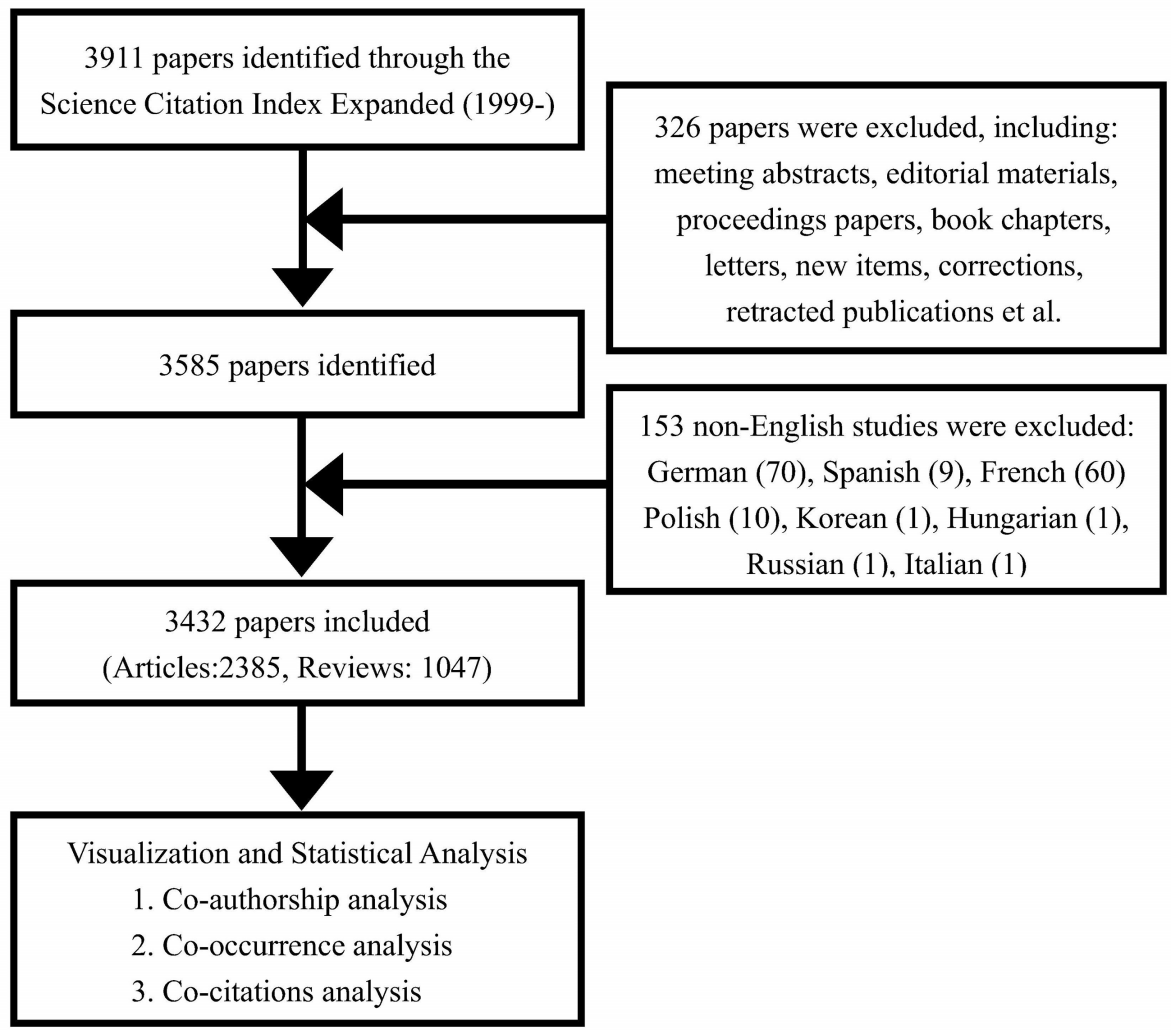
Flowchart of the literature searching and screening in the study.

The Web of Science Core Collection (WoSCC) is an optimal database with more than 10,000 high-quality journals and is also the most commonly used database in previous bibliometric studies (32, 33). In this study, the Science Citation Index-Expanded of the WoSCC was selected as our database. To avoid data bias, two researchers independently conducted the literature search for original articles and reviews on May 1, 2022. The searching terms are as follows: #1: Topic (TS)=(Immunotherapy OR Immunotherapies OR immunotherapeutic) OR Author Keywords (AK)=(Immunotherapy OR Immunotherapies OR immunotherapeutic); #2: TS=(renal OR kidney) NEAR/2 (cancer* OR tumor* OR tumour* OR oncology OR neoplasm* OR carcinoma*) OR AK=(renal OR kidney) NEAR/2 (cancer* OR tumor* OR tumour* OR oncology OR neoplasm* OR carcinoma*); Final data source: #1 AND #2. The period of study was from 1^st^ January, 2002 to 31^th^ December, 2021 and the language was restricted to English. The study flowchart is shown in **Figure 1**.

### Visualization and statistical analysis

Microsoft Office Excel 2019 (Microsoft, Redmond, WA, USA) was the main software used to analyze the data from the WoSCC and to construct a polynomial regression model to predict the number of publications and total citations in 2022. In addition, the indicators, which included the Hirsch index (H-index), impact factor (IF), and quartile in the category of journals, were also collected and analyzed through Microsoft Office Excel 2019. The H-index, proposed by Jorge Hirsch (34), is a mixed quantitative index, and it can be used to evaluate the number and level of academic output of researchers. The higher a researcher’s H-index, the greater the influence of his/her article. Furthermore, in many previous studies, the H-index was also applied to evaluate the productivity and academic status of countries, organizations, or journals (35–37). The Bibliometrix package in R software (Version 4.0.3) and an online bibliometric analysis platform (http://bibliometric.com/) were used to perform the collaboration among countries/regions. Visualization was mainly performed through VOSviewer and CiteSpace.

VOSviewer is widely used bibliometric visualization software to conduct network visualization maps and knowledge structure (38). The network visualization map, overlay visualization map, and density visualization map are the three main visual maps that VOSviewer provides. In this study, VOSviewer (Version 1.6.16) was utilized to conduct co-authorship analysis of country/author/institution, co-citation analysis of journal, and author keyword co-occurrence analysis. The options and settings of VOSviewer are displayed in **Supplementary Table S1**.

CiteSpace V (Version 5.8.R3) is another popular visualization tool that was developed by Professor Chaomei Chen (39–41). It was mainly applied to perform the visualization map of co-citation analysis of references/authors and to detect the keywords/references with the strongest citation bursts in this study. In addition, a dual-map overlay of journals was also created by CiteSpace V. The parameters included in CiteSpace were as follows: time span (2002–2021), year per slice (1 year), node type (reference, cited author, and cited journal), selection criteria (top 50 per slice), and pruning methods (minimum spanning tree (MST) and pruning sliced networks).

## Results

### Analysis of annual publications and citation trends

**Figure 2:**
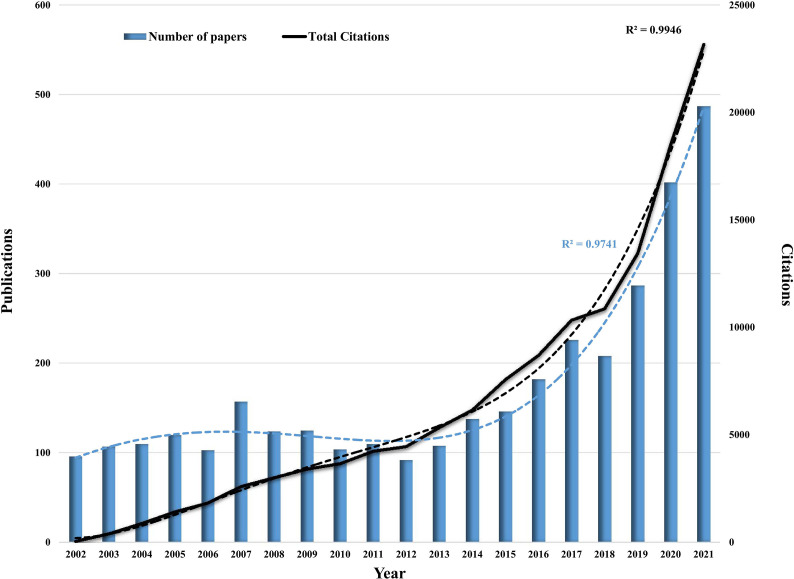
Global trend of publications and total citations on RCC immunotherapy from 2002 to 2021. The blue and black dotted lines represent the trend-fitted curves using polynomial regression model. The correlation coefficients (R^2^) are displayed in the figure.

A total of 3,432 publications in the field of RCC immunotherapy were collected after a thorough search. Regarding the search data, the sum of the times that all publications were cited was 135,782, and the average number of citations per item (ACI) was 39.56. The H-index of all papers was 147. As shown in **Figure 2**, between 2002 and 2012, the growth rate of the annual number of papers in RCC immunotherapy varied. After 2012, the annual number of relevant papers grew rapidly, reaching a peak in 2021 (487 papers). Through data fitting, a statistically significant relationship between publications and the published year (correlation coefficient R^2^ = 0.9741) became clear. Correspondingly, the curve of annual citations had shown a steady increase since 2002 and reached a peak in 2021 with 23,169 citations (R^2^ = 0.9946). According to the fitting curve in **Figure 2**, the annual number of publications and citations concerning RCC immunotherapy will be 611 and 28,688, respectively, in 2022.

### Contribution of active countries/regions

**Figure 3:**
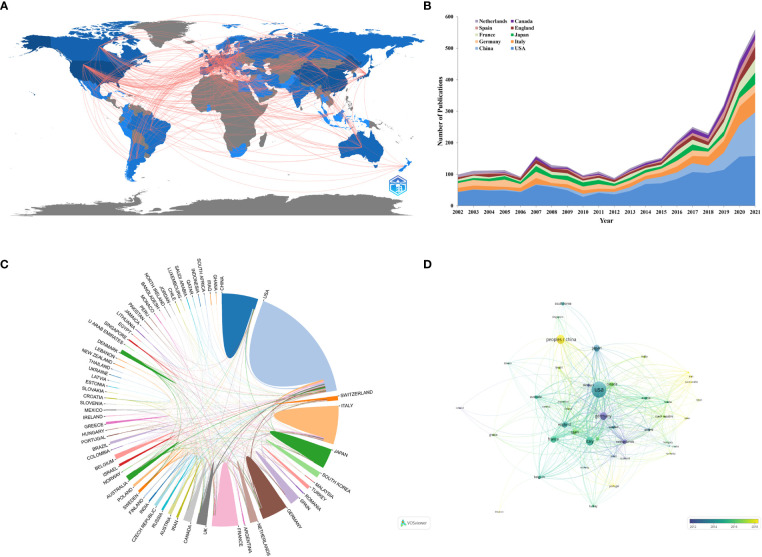
**(A)** Geographic distribution map based on the total publications of different countries/regions. **(B)** The changing trend of the annual publication quantity in the top 10 countries/regions from 2002 to 2021. **(C)** The international collaborations visualization map of countries/regions. **(D)** The countries/regions citation overlay visualization map generated by using VOSviewer. Each node means a country/region, and the size of node indicates the number of publications. The connection between the nodes represents the citation relationship, and the thickness of the connection lines indicates citation strength.

A total of 73 countries/regions were included in the study. **Figure 3A** shows the geographical distribution map of the RCC immunotherapy study. It can be observed that studies about RCC immunotherapy were mainly reported from the countries in North America, Europe, and Asia. The annual number of publications of the top 10 productive countries/regions is displayed in **Figure 3B**, showing that the number of publications concerning RCC immunotherapy retains a swift growth. The top 10 productive countries/regions as regards the number of publications are listed in **Table 1**. Among them, the United States is the leading country in this field with 1,431 papers, accounting for more than 41% of all papers included. It is noteworthy that of the top 10 productive countries, China is the only country with an ACI lower than 20 even though China ranks second in the number of publications.

**Table 1:** Top 10 productive countries/regions related to RCC immunotherapy research.

Rank	Country	Counts	% of 3432	H-index	ACI	TLS
1	USA	1431	41.696	131	61.78	748
2	China	444	12.937	40	19.01	114
3	Italy	399	11.626	50	25.13	417
4	Germany	324	9.441	58	39.95	363
5	Japan	255	7.43	47	27.73	166
6	France	241	7.022	56	50.68	380
7	England	201	5.857	46	39.88	322
8	Canada	127	3.7	38	49.71	251
9	Spain	127	3.7	33	30.15	293
10	Netherlands	125	3.642	41	47.09	202

ACI, Average Citations per Item; TLS, Total Link Strength.

For the collaboration analysis of countries/regions, an online bibliometric platform and VOSviewer were utilized to construct the co-authorship network map of countries. The international cooperation map indicated the extensive cooperation among countries (**Figure 3C** **)**. The United States is the cooperation center in this field, having the closest relationship with Italy, France, and China. As shown in **Figure 3D**, the top three countries with the highest total link strength (TLS) are ranked as follows: the United States (TLS = 748), Italy (TLS = 417), and France (TLS = 380). Germany was the first to study RCC immunotherapy, with an average publication year of 2011.89. It is noteworthy that China has become active in recent years with an average publication year of 2018.15, which started much later than most productive countries.

### Contribution of productive organizations and funding agencies

**Figure 4:**
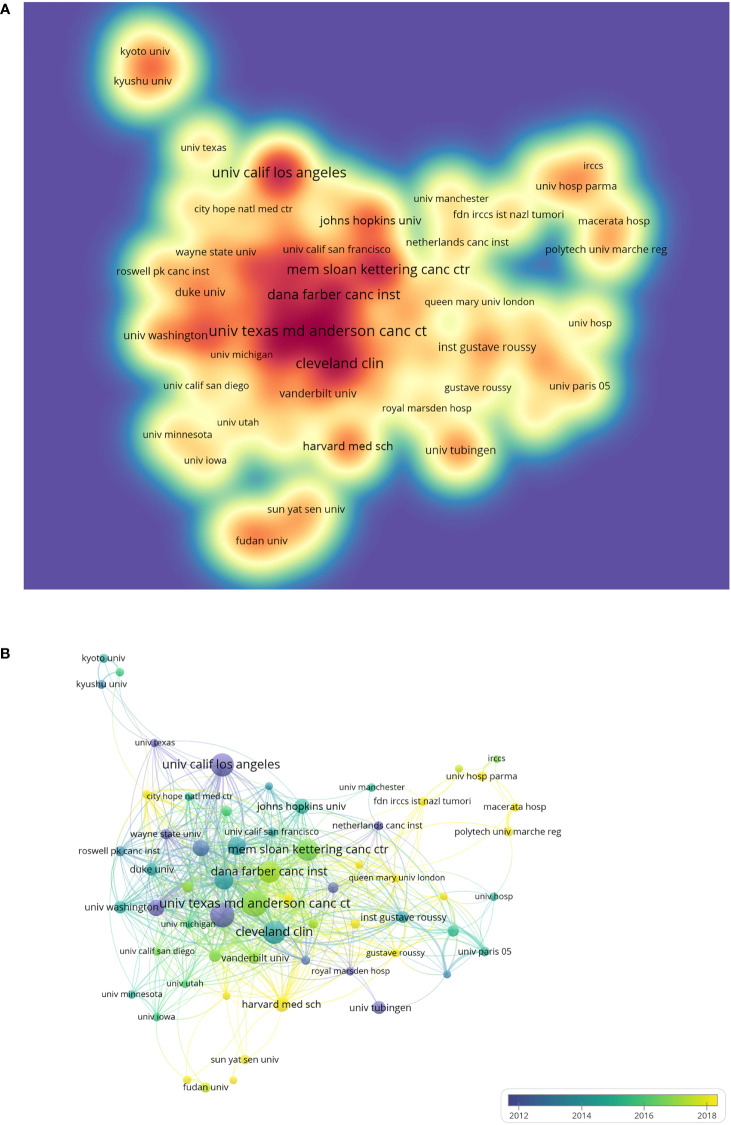
**(A)** The spectral density map of organizations was performed with VOSviewer. The deeper the color of the node, the more documents the organization published. **(B)** The overlay visualization map of organizations’ collaborations based on VOSviewer. The purple nodes represented the early institutions that participated in the research in this field, while the yellow nodes reflected the later organizations.

A total of 3,756 organizations have participated in the RCC immunotherapy study. From the results of **Table 2**, it can be observed that the top 10 productive organizations are all from the United States. The University of Texas MD Anderson Cancer Center ranks first in terms of the number of papers (N = 100), followed by the National Cancer Institute (N = 91), Cleveland Clinic (N = 84), and University of California, Los Angeles (N = 84). Total citations is an important index for measuring the international influence of institutions. As displayed in **Table 2**, the Memorial Sloan Kettering Cancer Center, Dana-Farber Cancer Institute, and Beth Israel Deaconess Medical Center are the top three organizations with the highest total citations. Only organizations with a minimum of 20 papers were included and visualized in the spectral density map (**Figure 4A**). The top three organizations with the highest TLS are the Dana-Farber Cancer Institute, Beth Israel Deaconess Medical Center, and Memorial Sloan Kettering Cancer Center. The overlay visualization map of organizations’ collaboration is shown in **Figure 4B**. Organizations in the United States or Germany began RCC immunotherapy studies earlier than those in China. Organizations in China, such as Sun Yat-sen University and Fudan University, have become active in recent years and have published more important papers in this field.

**Table 2:** The top 10 most productive organizations and funding agencies related to RCC immunotherapy research.

Rank	Organizations	Countries	Counts	TLS	Total Citations	Funding Agencies	Countries	Counts
1	University of Texas MD Anderson Cancer Center	USA	100	370	9202	United States Department of Health Human Services	USA	476
2	National Cancer Institute	USA	91	226	7074	National Institutes of Health	USA	475
3	Cleveland Clinic	USA	84	352	5313	National Cancer Institute	USA	376
4	University of California, Los Angeles	USA	84	154	7158	National Natural Science Foundation of China	China	192
5	Memorial Sloan Kettering Cancer Center	USA	79	371	15206	Bristol Myers Squibb	USA	98
6	Dana-Farber Cancer Institute	USA	75	479	14436	Pfizer	USA	75
7	Beth Israel Deaconess Medical Center	USA	64	402	12719	Novartis	Switzerland	67
8	Mayo Clinic	USA	61	185	9463	European Commission	European Commission	66
9	Harvard University	USA	53	197	6113	Ministry of Education Culture Sports Science And Technology	Japan	58
10	University of Pittsburgh	USA	53	176	2828	Roche Holding	Switzerland	44

TLS, Total Link Strength.

The top 10 funding agencies are also summarized in **Table 2**. Among them, half of the total funding agencies are from the United States, showing the United States’ strong economic foundation and support for scientific study. The United States Department of Health and Human Services ranked first with 476 papers, followed by the National Institutes of Health (N = 475) and the National Cancer Institute (N = 376).

### Contribution of active authors

**Figure 5:**
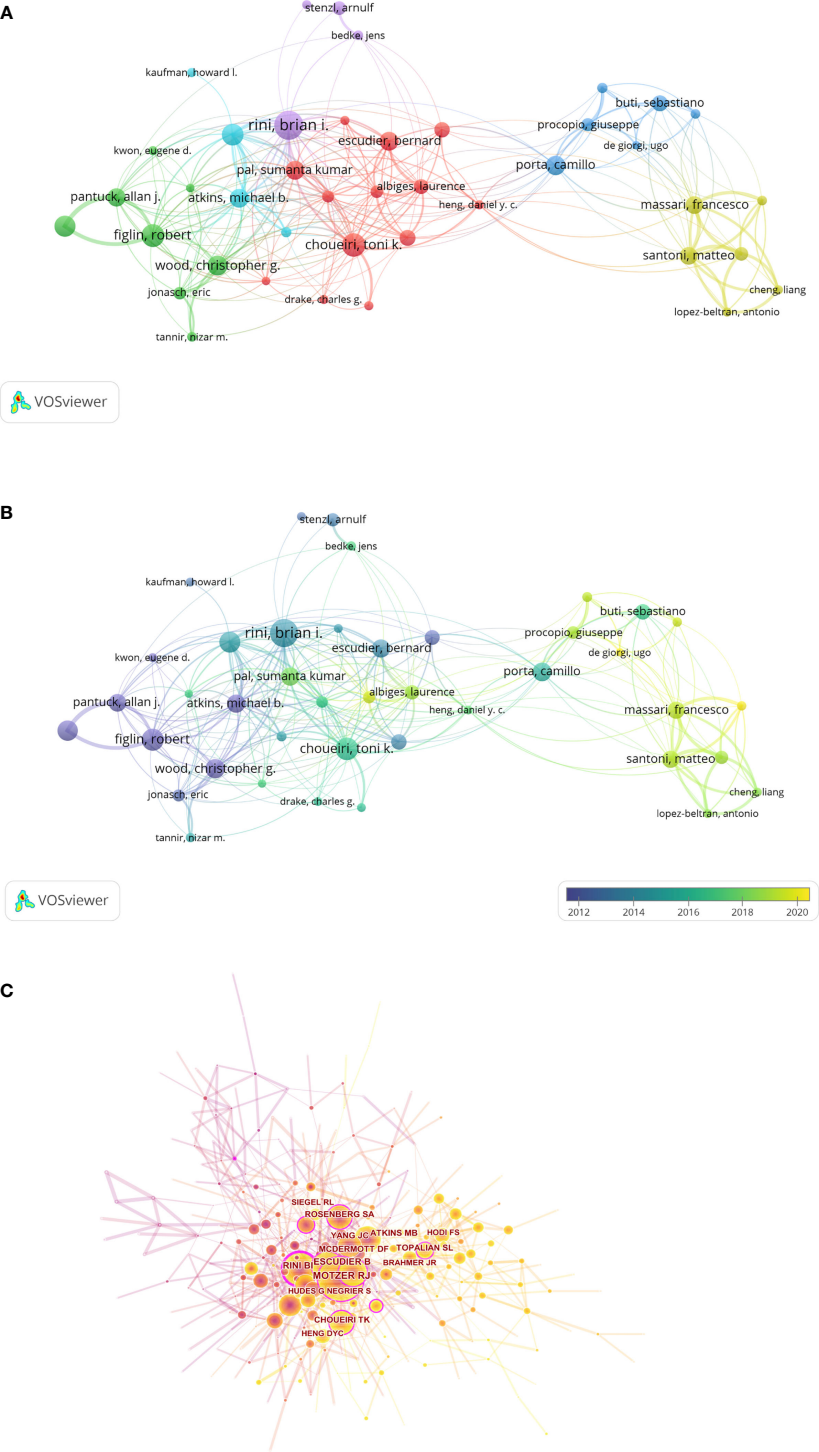
The network visualization map **(A)** and overlay visualization map **(B)** of author co-authorship analysis conducted by VOSviewer. **(C)** The visualization map of co-cited authors carried on CiteSpace.

Of all authors who participated in the studies on RCC immunotherapy, the 10 most productive authors and the top 10 authors with the largest centrality are summarized in **Table 3**. It is easy to deduce that eight of the top 10 authors are from the United States, and the two remaining authors are from Italy and France. Among them, the top three authors with the most papers are Brian I. Rini (N = 60), Toni K. Choueiri (N = 47), and Robert Figlin (N = 46). It is noteworthy that Brian I. Rini is also the top co-cited author with the largest centrality of 0.32. The authors with a minimum number of 15 documents are shown in **Figure 5A**, and when combined with the overlay visualization map of author co-authorship analysis (**Figure 5B**), it can be observed that the authors in the green cluster are considered pioneers in the field of RCC immunotherapy, whereas the authors in the blue and yellow clusters began to publish papers in recent years. In addition, close collaboration and communication among different clusters are lacking. As shown in the map of co-cited authors (**Figure 5C**), Brian I. Rini, Robert J. Motzer, and Toni K. Choueiri are the top three co-cited authors in this analysis, showing their dominance in this field.

**Table 3:** The 10 most productive authors and the top 10 authors with largest centrality in the field of RCC immunotherapy.

Rank	Author	Country	Counts	Total Citations	H-index	TLS	Co-Cited Author	Country	Total Citations	TLS	Centrality
1	Rini, Brian I.	USA	60	4474	26	550	Rini, Brian I.	USA	1868	132263	0.32
2	Choueiri, Toni K.	USA	47	1585	26	509	Motzer Robert J	USA	5065	300035	0.16
3	Figlin, Robert	USA	46	4519	28	374	Choueiri Toni K	USA	1235	83538	0.16
4	Mcdermott, David F.	USA	43	2589	26	435	Mcdermott David F	USA	872	62732	0.15
5	Belldegrun, Arie S.	USA	42	4102	29	326	Powles T	UK	371	30888	0.15
6	Wood, Christopher G.	USA	39	1752	20	303	Amato Robert J	USA	324	23158	0.15
7	Porta, Camillo	Italy	38	649	16	306	Escudier, Bernard	France	1546	99947	0.13
8	Atkins, Michael B.	USA	37	2828	23	355	Rosenberg Steven A	USA	945	87075	0.13
9	Escudier, Bernard	France	37	2750	25	352	Simons JW	USA	91	7776	0.12
10	Pal, Sumanta Kumar	USA	37	904	16	301	Topalian Suzanne L	USA	645	51850	0.11

TLS, Total Link Strength.

### Analysis of influential journals and co-cited journals

**Figure 6:**
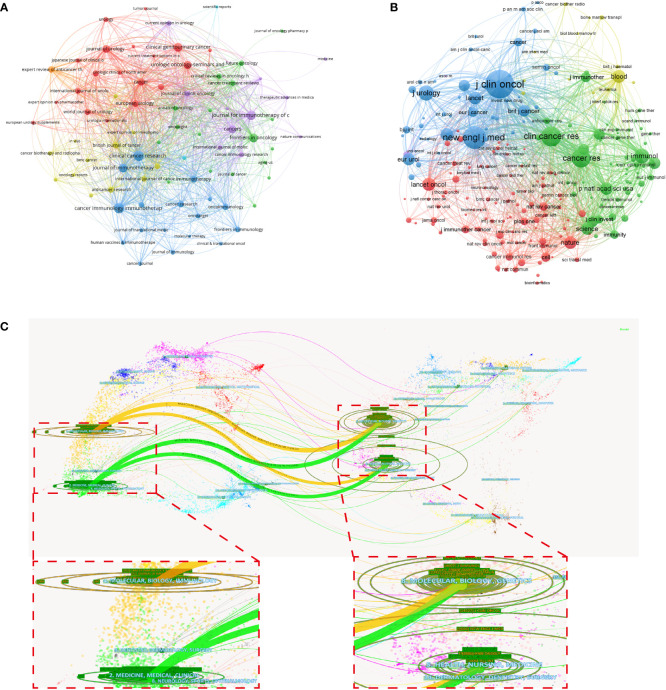
The network visualization maps of citing journals **(A)** and co-cited journals **(B)** were produced by VOSviewer. **(C)** A dual-map overlap of journals on RCC immunotherapy carried out by CiteSpace.

More than 700 journals were assessed in this study, with *Cancer Immunology Immunotherapy* (N = 102, IF = 6.968, Q1), *Clinical Cancer Research* (N = 99, IF = 12.531, Q1), and *Journal for Immunotherapy of Cancer* (N = 85, IF = 13.751, Q1) as the top three journals with most publications (**Table 4**). Among the top 10 most productive journals, *Clinical Cancer Research* has the highest H-index (51) and total citations (8,585), whereas *Journal for Immunotherapy of Cancer* has the highest IF. In addition, all of the top 10 co-cited journals shown in **Table 4** are cited more than 2,800 times, with *Journal of Clinical Oncology* (14,539 times) being the most cited. The network visualization maps of citing journals and co-cited journals were produced using by VOSviewer. As shown in **Figures 6A**, **B**, many journals co-occurred in both maps and have active citation relationships.

**Table 4:** Top 10 productive journals and co-cited journals in the field of RCC immunotherapy.

Rank	Journals	Country	Counts	IF(2020)	JCR(2020)	H-index	Total Citations	Co-cited journals	IF(2020)	JCR(2020)	Total citations
1	Cancer Immunology Immunotherapy	USA	102	6.968	Q1	35	3528	Journal of Clinical Oncology	44.544	Q1	14539
2	Clinical Cancer Research	USA	99	12.531	Q1	51	8585	New England Journal of Medicine	91.245	Q1	9607
3	Journal For Immunotherapy of Cancer	UK	85	13.751	Q1	28	3002	Clinical Cancer Research	12.531	Q1	7090
4	Clinical Genitourinary Cancer	USA	72	2.872	Q3/Q4	16	757	Cancer Research	12.701	Q1	6273
5	Frontiers in Oncology	Switzerland	71	6.244	Q2	12	753	Journal of Urology	7.45	Q1	4188
6	Journal of Immunotherapy	USA	71	4.456	Q2	30	3056	Journal of Immunology	5.422	Q2	4008
7	Cancers	Switzerland	61	6.639	Q1	11	428	Blood	22.113	Q1	3076
8	Urologic Oncology-Seminars and Original Investigations	Netherlands	61	3.498	Q2/Q3	17	864	lancet oncology	41.316	Q1	3006
9	Journal of Urology	USA	56	7.45	Q1	36	4114	Annals of Oncology	32.976	Q1	2889
10	Cancer	USA	48	6.86	Q1	29	3170	Proceedings of The National Academy of Sciences of The United States of America	11.205	Q1	2851

IF, Impact Factor; JCR, Journal Citation Reports.

A dual-map overlay of journals, generated by CiteSpace, was applied to portray the topic distribution of scientific journals. As displayed in **Figure 6C**, the citation connections between citing and co-cited journals were indicated by four main color lines. All of the paths indicated that the studies published in Molecular/Biology/Genetics and Health/Nursing/Medicine journals are usually cited by Molecular/Biology/Immunology journals or Medicine/Medical/Clinical journals.

### Analysis of references and co-cited references

Reference analysis was conducted in this study to understand the development of RCC immunotherapy research. Therefore, the references with the most citations were analyzed, and CiteSpace was utilized to visualize the reference co-citation network. The top 10 cited and co-cited references are summarized in **Tables 5**, **6**. The most cited reference is the article published by Suzanne L. Topalian (2012) (42) in the *New England Journal of Medicine*, with 8,208 citations, followed by Julie R. Brahmer (2012) (43) and Julie R. Brahmer (2010) (44). From the results in **Table 6**, the top three co-cited references were all published by Robert J. Motzer (21, 45, 46).

**Table 5:** Top 10 cited papers **concerning** the research of RCC immunotherapy.

Title	Journals	First author	Year	Citations
Safety, Activity, and Immune Correlates of Anti-PD-1 Antibody in Cancer	New England Journal of Medicine	Topalian Suzanne L	2012	8208
Safety and Activity of Anti-PD-L1 Antibody in Patients with Advanced Cancer	New England Journal of Medicine	Brahmer Julie R	2012	5217
Phase I Study of Single-Agent Anti-Programmed Death-1 (MDX-1106) in Refractory Solid Tumors: Safety, Clinical Activity, Pharmacodynamics, and Immunologic Correlates	Journal of Clinical Oncology	Brahmer Julie R	2010	2036
Mechanism-driven biomarkers to guide immune checkpoint blockade in cancer therapy	Nature Reviews Cancer	Topalian Suzanne L	2016	1332
PD-L1 Expression as a Predictive Biomarker in Cancer Immunotherapy	Molecular Cancer Therapeutics	Patel Sandip Pravin	2015	1181
Renal cell carcinoma	Lancet	Rini Brian I	2009	1046
Loss of tumor suppressor PTEN function increases B7-H1 expression and immunoresistance in glioma	Nature Medicine	Parsa Andrew T	2007	970
Cytokines in cancer pathogenesis and cancer therapy	Nature Reviews Cancer	Dranoff G	2004	951
Renal cell carcinoma	Nature Reviews Disease Primers	Hsieh James J	2017	907
The evolving landscape of biomarkers for checkpoint inhibitor immunotherapy	Nature Reviews Cancer	Havel Jonathan J	2019	901

**Table 6:** Top 10 co-cited references involved in the research of RCC immunotherapy.

Title	First author	Year	Citations	Journals	IF (2020)
Nivolumab versus Everolimus in Advanced Renal-Cell Carcinoma	Robert J Motzer	2015	615	New England Journal of Medicine	91.245
Sunitinib versus interferon alfa in metastatic renal-cell carcinoma	Robert J Motzer	2007	461	New England Journal of Medicine	91.245
Nivolumab plus Ipilimumab versus Sunitinib in Advanced Renal-Cell Carcinoma	Robert J Motzer	2018	400	New England Journal of Medicine	91.245
Temsirolimus, interferon alfa, or both for advanced renal-cell carcinoma	Gary Hudes	2007	354	New England Journal of Medicine	91.245
Safety, activity, and immune correlates of anti-PD-1 antibody in cancer	Suzanne L Topalian	2012	340	New England Journal of Medicine	91.245
Sorafenib in advanced clear-cell renal-cell carcinoma	Bernard Escudier	2007	332	New England Journal of Medicine	91.245
Results of treatment of 255 patients with metastatic renal cell carcinoma who received high-dose recombinant interleukin-2 therapy	G Fyfe	1995	310	Journal of Clinical Oncology	44.54
Pembrolizumab plus Axitinib versus Sunitinib for Advanced Renal-Cell Carcinoma	Brian I Rini	2019	298	New England Journal of Medicine	91.245
Nephrectomy followed by interferon alfa-2b compared with interferon alfa-2b alone for metastatic renal-cell cancer	R C Flanigan	2001	282	New England Journal of Medicine	91.245
Radical nephrectomy plus interferon-alfa-based immunotherapy compared with interferon alfa alone in metastatic renal-cell carcinoma: a randomised trial	G H Mickisch	2001	260	Lancet	79.321

IF, impact factor.

**Figure 7:**
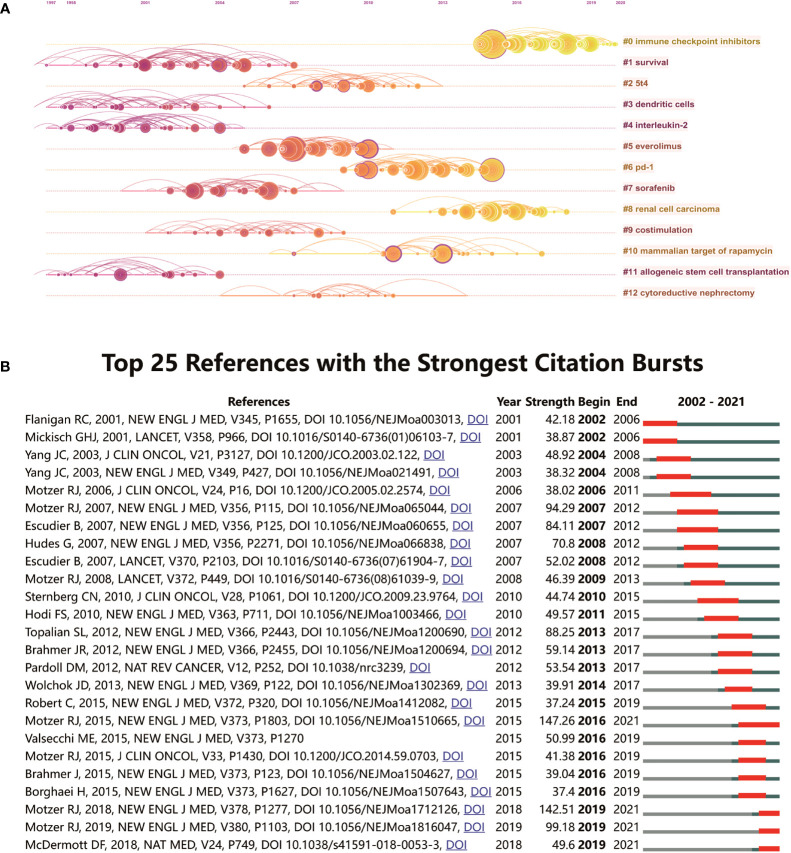
**(A)** CiteSpace visualization map of timeline view of co-citation references analysis. **(B)** CiteSpace visualization map of top 25 references with the strongest citation bursts from 2002 to 2021.

As shown in **Supplementary Figure S1**, the reference co-citation network map is composed of 536 nodes, which can be grouped into 13 subclusters. The modularity Q and the mean silhouette S were higher than 0.75, showing a significant cluster result and a good homogeneity effect. Simultaneously, the timeline view of co-cited references visually shows the changing trend of research topics over time (**Figure 7A**). It can be observed that #1 survival, #3 dendritic cells, #4 interleukin-2, and #11 allogeneic stem cell transplantation are the early research topics in this field. Clusters #0 immune checkpoint inhibitors, #6 pd-1, #8 renal cell carcinoma, and #10 mammalian target of rapamycin are located at the line’s rightmost end, demonstrating the current new research foci in this field. Reference citation bursts were applied to show the popularity and importance over time of references in this field. From the results of **Figure 7B**, we can summarize that the publications of R. C. Flanigan (2001) (47) and G. H. J. Mickisch (2001) (48) are the references with the earliest citations bursts. Meanwhile, R. J. Motzer (2015) (45), R. J. Motzer (2018) (21), R. J. Motzer (2019) (49) and D. F. McDermott (2018) (50) have current emergence of strong citation references.

### Analysis of keyword co-occurrence

**Figure 8:**
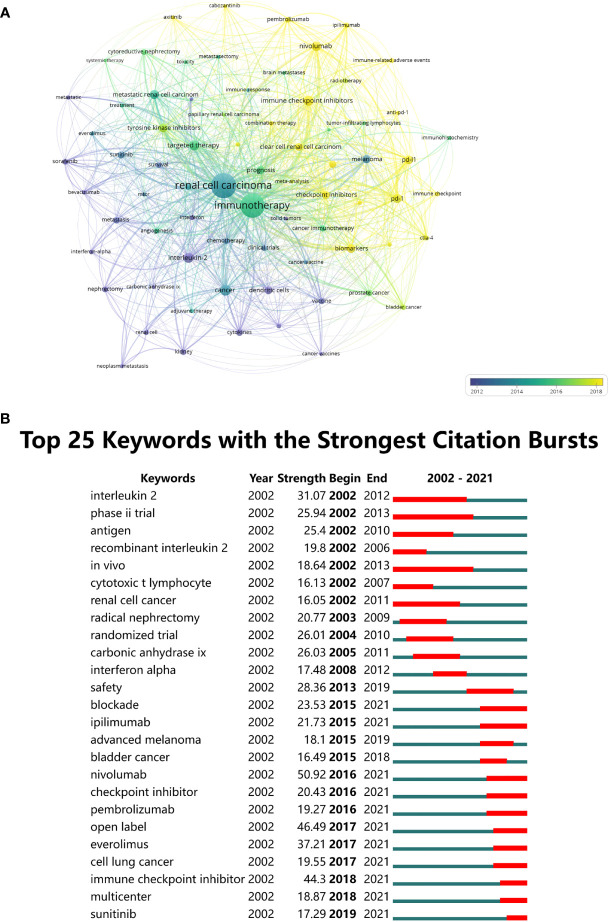
**(A)** The time-overlay visualization map of the co-occurrence keywords generated by using VOSviewer. **(B)** CiteSpace visualization map of top 25 keywords with the strongest citation bursts of publications in the field of RCC immunotherapy from 2002 to 2021.

After the synonymous keywords were merged and meaningless keywords removed, VOSviewer software was applied to create the overlay visualization map of keywords. There were 4,539 keywords included, and 73 keywords emerged with a minimum of 20 occurrences (**Figure 8A**). Among them, the top 20 co-occurrence author keywords with the highest frequency in this study are listed in **Table 7**. It can be observed that “immune checkpoint inhibitors”, “targeted therapy”, “pd-1”, “pd-l 1”, and “nivolumab” are the keywords that have occurred in recent years; in other words, these keywords seem to represent the current research frontiers (**Figure 8A**). The top 25 keywords with the strongest citation bursts were also detected through CiteSpace (**Figure 8B**), and when combined with the most frequent keywords in **Figure 8A**, the keywords related to RCC immunotherapy with ongoing citation bursts until 2021 were “blockade”, “ipilimumab”, “nivolumab”, “checkpoint inhibitor”, “pembrolizumab”, “open label”, “everolimus”, “immune checkpoint inhibitor”, “multicenter”, and “sunitinib”. These were the keywords that we mainly focused on because of their effect in identifying the frontiers of RCC immunotherapy research.

**Table 7:** Top 20 co-occurrence keywords involved in the research of RCC immunotherapy.

Rank	Keywords	Occurrences	TLS	Rank	Keywords	Occurrences	TLS
1	renal cell carcinoma	1286	5641	11	tyrosine kinase inhibitors	123	715
2	immunotherapy	1200	5625	12	biomarkers	122	573
3	cancer	207	1021	13	prognosis	119	517
4	interleukin-2	178	786	14	melanoma	116	617
5	immune checkpoint inhibitors	175	916	15	checkpoint inhibitors	104	605
6	targeted therapy	173	811	16	dendritic cells	98	592
7	nivolumab	154	851	17	clear cell renal cell carcinoma	98	536
8	pd-1	147	878	18	sunitinib	83	433
9	metastatic renal cell carcinoma	146	663	19	tumor microenvironment	82	425
10	pd-l1	128	793	20	metastasis	74	320

TLS, Total Link Strength.

## Discussions

### General information

Different from reviews or meta-analysis, bibliometric analysis has unique advantages in summarizing the development trend of specific research fields and detecting the research focus. This is the first study to perform a knowledge structure and to analyze the next potential research frontiers in the field of RCC immunotherapy study by using the bibliometric method.

RCC is one of the most common malignancies in both men and women, and its treatment is also a global concern health concern. In the past 20 years, as shown in **Figure 2**, great progress has been made in the field of RCC immunotherapy. However, concerning the analysis of countries, there are only 73 countries/regions included in this study, and notably, less than half of the countries published more than 10 papers. It is noteworthy that although China has been participating actively in this field with more than 400 papers, its ACI is the lowest among the top 10 countries. Therefore, the two aspects that should be focused on to change the status quo are as follows: 1) increasing cooperation and exchange with other countries, especially the United States, Italy, and Germany, and 2) paying close attention to scientific innovations to improve the quality of publications.

The top 10 most productive organizations are from the United States. This might explain why the United States contributed the most to the study of RCC immunotherapy. These results implied an imbalance of global academic resources, and the establishment of world-class scientific organizations is the key foundation in promoting the national academic status. In addition, funding support also plays an important role in scientific research, which was also confirmed in this study (**Table 2**).

Of the top 10 prolific authors, Brian I. Rini is the most prolific author with 60 papers and 4,474 citations in this field. Moreover, among the top co-cited authors, it is obvious that Brian I. Rini is also ranked first as regards centrality, followed by Robert J. Motzer and Toni K. Choueiri. Brian I. Rini is an oncologist at Vanderbilt University Medical Center, and he is famous for his contributions to exploring the treatment and immunotherapy mechanisms of RCC (51). Robert J. Motzer has led many clinical trials on patients with kidney carcinoma and has published many high-level articles in *New England Journal of Medicine*. Toni K. Choueiri published a well-known review titled “Systemic Therapy for Metastatic Renal-Cell Carcinoma” in *New England Journal of Medicine*, which systemically summarized the current first-line or second-line therapeutic schedules, as well as surgery strategies for RCC, and constructively put forward the future research directions for RCC treatments (52). Therefore, the aforementioned articles provided reliable reference value for researchers in this field.

Among the top 10 productive journals, *Cancer Immunology Immunotherapy* published the most papers related to RCC immunotherapy, showing its core role in this field, and more significant findings are more likely to be published in this journal. Except for *Journal of Immunology*, all of the co-cited journals in **Table 4** are located in Q1, demonstrating the importance of RCC immunotherapy in future research.

### Knowledge base

Co-citation analysis is an effective method to evaluate the degree of connection among papers (37, 53). It is generally believed that the higher the citation frequency of an article, the more meaningful it is in this field. The top 10 co-citation references shown in **Table 6** are all well-known clinical trials published in top-ranked journals. These papers summarized the discovery and development of RCC immunotherapy from cytokines to target therapy and then to ICI-based immunotherapy.

Until 2004, the cytokines IL-2 and IFN-α were considered the only therapeutic drugs that target mRCC. A high-dose IL-2 regimen was also approved by the FDA in 1992 for the treatment of mRCC. Fyfe et al. (54) performed a clinical trial in 1995 to identify the safety and efficacy of high-dose IL-2 in 255 patients with mRCC. Although their results showed that patients with mRCC benefited from high-dose IL-2, severe complications and metastatic diseases still occurred. IFN-α is another cytokine used for treating mRCC and has less severe toxicities, although its overall treatment effect is unsatisfactory. Clinical studies conducted by Flanigan et al. (47) and Mickisch et al. (48) demonstrated that radical nephrectomy before IFN-α treatment can improve the OS and delay the time of disease progression in patients with mRCC. As demonstrated, the treatment options for mRCC are limited. Although IL-2 and IFN-α have suboptimal efficacy and high incidence of toxicity, before 2004, they were the only treatment for patients with mRCC. IL-2 and IFN-α were the beginning of RCC immunotherapy, promoting the development of targeted therapy and combination therapy.

With the deepening of the research on the mechanism of RCC, drugs targeting the pathogenesis of RCC, such as VEGF inhibitors or Mechanistic Target of Rapamycin Complex 1 (mTORC1) inhibitors, have also been continuously developed and applied. Sorafenib is the first VEGF inhibitor drug approved by the FDA in 2005 for treating RCC. Subsequently, sunitinib, pazopanib, and axitinib received FDA approval and are being widely used in the clinical setting. There have been many phase 3 clinical trials that determined the effects of targeted drugs on disease progression and OS in patients with advanced RCC. Motzer et al. (46) studied the curative effects of sunitinib and IFN-α in patients with mRCC, and their results showed that the median progression-free survival (11 vs. 5 months) and objective response rate (31% vs. 6%) were higher in patients with mRCC who were treated with sunitinib than in those who received IFN-α. At the same time, Escudier et al. (55) published a randomized, double-blind, phase 3 study on sorafenib in the treatment of advanced ccRCC, which demonstrated that sorafenib, compared with placebo, can improve the median progression-free survival of patients with advanced ccRCC. Nevertheless, they observed adverse reactions such as hypertension. A few months after the publication of Escudier et al., another well-known randomized clinical trial was published by Hudes et al. (56). Their results showed that compared with IFN-α, temsirolimus might improve the OS of patients with advanced RCC. The aforementioned studies confirmed that the VEGF receptor and mTOR are important targets for the treatment of RCC, and many targeted drugs have also been developed and applied in the clinical setting, improving the OS and progression-free survival of patients. Moreover, sunitinib has become the standard control drug in RCC clinical studies (3).

However, the application of these targeted therapies is often limited by drug resistance. In 2012, Topalian et al. (42) assessed the antitumor activity and safety of an anti-PD-1 antibody (BMS-936558) and preliminarily demonstrated the role of anti-PD-1 in the treatment of RCC. Three years later, nivolumab (PD-1 inhibitor) became the first checkpoint inhibitor to receive FDA approval for RCC treatment. Since then, many clinical studies have proved the safety and efficacy of different ICIs in patients with RCC. The CheckMate 025 study (45) compared the safety and efficacy of nivolumab with those of everolimus in 821 patients with advanced RCC. As their results showed, nivolumab can provide better median OS and objective response than everolimus. Motzer et al. (21, 57) conducted a phase 3 trial to compare the therapeutic efficacy of nivolumab plus ipilimumab with that of sunitinib, and they found that the dual ICI group achieved better OS and progression-free survival than the sunitinib group. This study was the first to demonstrate that dual-ICI combination therapy is more promising than the combination of VEGF and mTOR inhibitor, and nivolumab plus ipilimumab was approved by the FDA as the first-line treatment of mRCC. Since then, many combination therapies have been proposed and confirmed, providing more immunotherapy choices for RCC treatment (18, 49).

### Emerging hotspots

The timeline view of co-cited references visualized the dynamic evolution and research hotspots of RCC immunotherapy. As shown in **Figure 7A**, the research focus has shifted from #1 survival, #3 dendritic cells, #4 interleukin-2, and #11 allogeneic stem cell transplantation to #2 5T4, #5 everolimus, #7 sorafenib, #9 costimulation, and #12 cytoreductive nephrectomy. Currently, clusters #0 immune checkpoint inhibitors, #6 pd-1, #8 renal cell carcinoma, and #10 mammalian target of rapamycin are the new research hotspots in this field.

Reference citation burst detection is a method to identify the references that are highly cited over a certain period. From the results presented in **Figure 7B**, there are four references with citation bursts to date. R. J. Motzer (2015) (45) is the reference with the strongest citation burst (burst strength 147.26, 2016–2021), followed by R. J. Motzer (2018) (21), R. J. Motzer (2019) (49) and D. F. McDermott (2018) (50). The rise and development of ICIs have brought RCC immunotherapy into a new stage. Nivolumab is the first ICI drug approved by the FDA for RCC treatment and has become one of the most representative drugs in RCC immunotherapy. In the following years, nivolumab or nivolumab-based combination therapy has been widely applied in many clinical studies. Currently, combination therapy may be one of the optimal choices for the immunotherapy of RCC. The first combination therapy implemented in this field was nivolumab-based combination therapy in 2012 (58, 59). R. J. Motzer (2018) (21), R. J. Motzer (2019) (49) and D. F. McDermott (2018) (50) conducted significant trials of combination immunotherapy for RCC, and these studies confirmed that ICI-based combination therapy was more effective than sunitinib for RCC treatment. Undoubtedly, combination therapy is the current research hotspot of RCC immunotherapy, showing good therapeutic potential. The purpose of combination therapy is to improve therapeutic efficacy without affecting safety. However, because combination therapy may create more adverse effects, some clinical trials of ICI monotherapy are also being carried out simultaneously, which hope to find a better ICI therapy regimen as an alternative treatment with fewer adverse effects. Based on the results of D. F. McDermott (2018) (50), atezolizumab (PD-L1 inhibitor) exhibited a high response rate and was well tolerated, showing its excellent potential in ICI monotherapy.

Unfortunately, the efficacy of ICI-based therapy for solid tumors is still unsatisfactory (60–62). On the one hand, the response rate to ICI-based therapy is closely related to many factors such as the tumor mutational burden, indicating that ICI-based therapy may be not effective for most patients. On the other hand, ICI-based therapy may produce some toxic adverse effects. Moreover, there are currently no methods or ancillary tests to identify the precise group that can benefit from ICI-based therapy. Therefore, the development of new biomarkers for predicting the response rate to ICIs and selecting patients who can gain therapeutic benefits may be of great value for RCC immunotherapy.

“Keywords with citation bursts” were also analyzed through CiteSpace in this study. By combining **Figure 7A** with **Figure 8B**, one can observe that the bursting keywords were consistent with the current research hotspots analyzed above. Of these keywords, several bursting keywords such as ipilimumab, nivolumab, or pembrolizumab are designated as ICIs, which demonstrated that research on ICIs would still be the focus of this field in the future. It is noteworthy that everolimus and sunitinib were also bursting keywords, indicating that many clinical trials based on the combination therapy of ICIs and mTOR/VEGF inhibitors will be performed in the future. This type of combination therapy is expected to achieve better therapeutic effects and decreased toxic adverse effects through the combination of drugs with different mechanisms of action.

### Limitations

Some limitations should be noted in this study. First, the data in this study were collected from the WoSCC database, which means some relevant papers in other data sources may have been excluded. Second, the focus was only on papers published in English, and as a result, high-quality articles published in other languages may have been ignored, leading to selection bias. Last, recently published high-quality papers may not appear in our analysis due to the low citations.

## Conclusion

In summary, this is the first bibliometric analysis to outline the knowledge map of RCC immunotherapy from 2002 to 2021 and to predict the future research hotspots. In this analysis, the United States was the leading country with the most high-quality publications and was also the country with the most international communication and cooperation. The cooperation should be enhanced among organizations. Moreover, “immune checkpoint inhibitors” were the most important research hotspot, and the research on ICIs mainly included the following aspects, which may be the next research hotspots: combination therapy, ICI monotherapy, and the development of new predictive biomarkers. Research in this field will help us develop tailored treatment regimens and achieve precision medicine for specific patients with RCC.

## Data availability statement

The original contributions presented in the study are included in the article/**supplementary material**. Further inquiries can be directed to the corresponding authors.

## Author contributions

ZS, JK and HW conceived the study. KL and SZ collected the data and KL wrote the manuscript. JL and YZ analyzed the data. JK, HW and ZS revised and reviewed the manuscript. All authors contributed to the article and approved the submitted version.

## Funding

This study was supported by the Xiamen Medical and Health Guidance Project (3502Z20209069).

## Acknowledgements

We thank LetPub (www.letpub.com) for its linguistic assistance during the preparation of this manuscript.

## Conflict of interest

The handling editor YZ declared a shared affiliation with the authors JK and ZS at the time of review.

The remaining authors declare that the research was conducted in the absence of any commercial or financial relationships that could be constructed as a potential conflict of interest.

## Publisher’s note

All claims expressed in this article are solely those of the authors and do not necessarily represent those of their affiliated organizations, or those of the publisher, the editors and the reviewers. Any product that may be evaluated in this article, or claim that may be made by its manufacturer, is not guaranteed or endorsed by the publisher.
