# Supplementary material for: Knowledge mapping and research hotspots of immunotherapy in renal cell carcinoma: A text-mining study from 2002 to 2021
Source: Front Immunol. 2022 Jul 28;13:969217. doi: 10.3389/fimmu.2022.969217 (PMC9367473; doi:10.3389/fimmu.2022.969217)
Supplement: Supplementary file 1 [file DataSheet_1.docx]

Supplementary Material

1. **Supplementary Figures and Tables**

**1.1 Supplementary Figures**

**
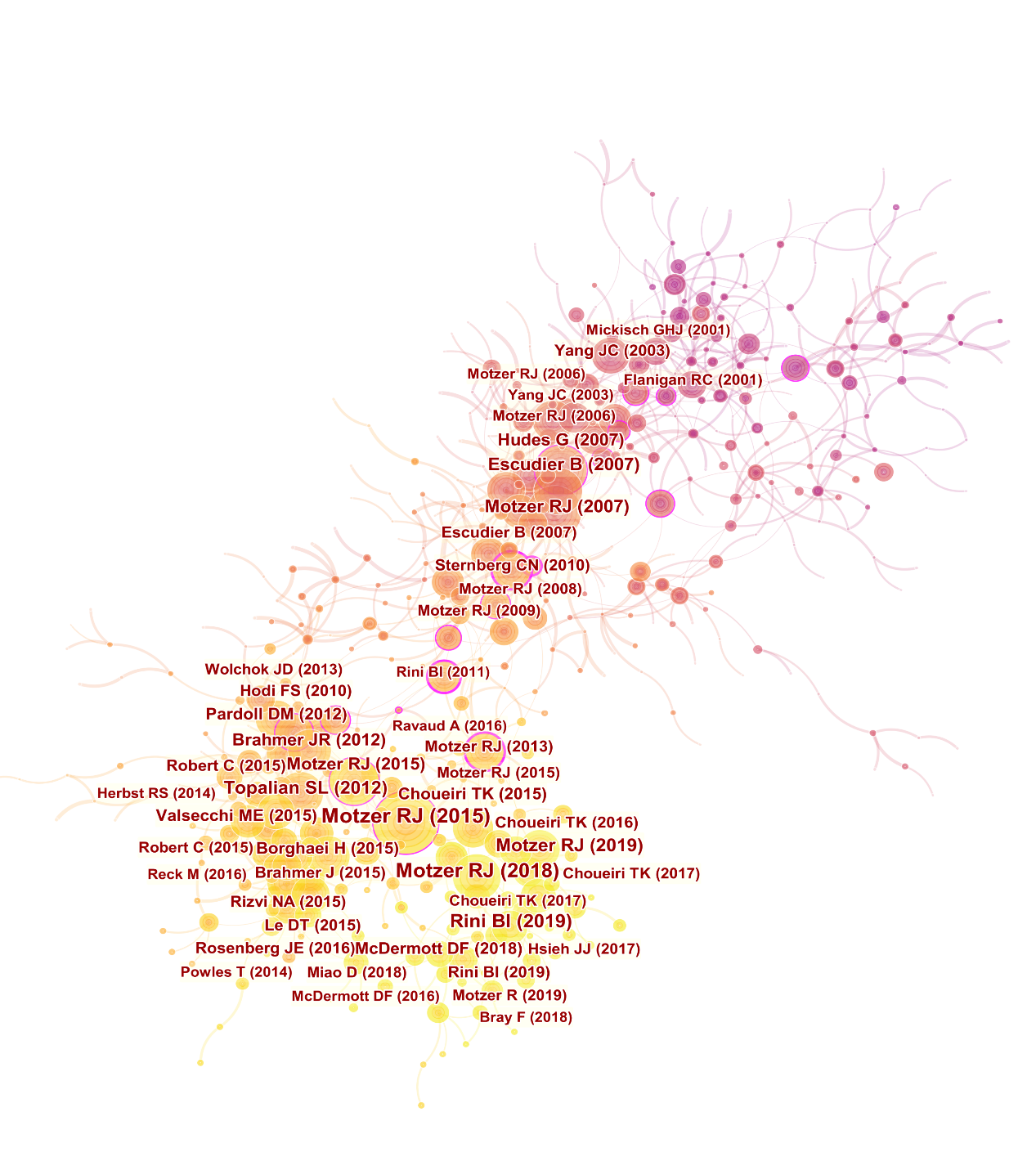
**

**Supplementary Figure S1** CiteSpace visualization map of Cluster view of co-citation references analysis.

## 1.2 Supplementary Tables

**Table S1. The options and settings of VOSviewer for RCC immunotherapy study.**

| Analysis | Unit of analysis | thresholds | Visualization methods |
| --- | --- | --- | --- |
| Co-authorship | Country/region | Minimum number of documents of a country: 5 | Overlay visualization |
|  | Author | Minimum number of documents of an author: 15 | Network visualization and Overlay visualization |
|  | Institution | Minimum number of documents of an institution: 20 | Density visualization and Overlay visualization |
| Citation | Journal | Minimum number of documents of a source: 10 | Network visualization |
| Co-citation | Journal | Minimum number of documents of a source: 100 | Network visualization |
| Co-occurrence | Author keywords | Minimum number of occurrences of a keyword: 20 | Overlay visualization |
